# Baseline energy and nutrient intakes of AU‐ARROW participants using a Food Frequency Questionnaire‐ Preliminary findings

**DOI:** 10.1002/alz.091119

**Published:** 2025-01-09

**Authors:** Malika G Fernando, Carolina B Castro, Samantha L Gardener, Stephanie Jane Fuller, Sharon L Naismith, Laura D Baker, Miia Kivipelto, Victor L Villemagne, Stuart Grieve, Paul A Yates, Nicola Armstrong, Kaarin J. Anstey, Hamid R. Sohrabi, Manohar Garg, Juliana Chen, Ralph N Martins

**Affiliations:** ^1^ Macquarie University, Sydney, NSW Australia; ^2^ Murdoch University, Murdoch, Western Australia Australia; ^3^ Alzheimer’s Research Australia, Perth, Western Australia Australia; ^4^ Edith Cowan University, Perth, Western Australia Australia; ^5^ The University of Sydney, Sydney, NSW Australia; ^6^ Wake Forest University, Winston‐Salem, NC USA; ^7^ Karolinska Institutet, Stockholm Sweden; ^8^ University of Eastern Finland, Kuopio Finland; ^9^ University of Melbourne, Melbourne, VIC Australia; ^10^ Austin Health, Melbourne, VIC Australia; ^11^ University of Pittsburgh, Pittsburgh, PA USA; ^12^ Curtin University, Perth, Western Australia Australia; ^13^ University of New South Wales, Sydney, NSW Australia

## Abstract

**Background:**

Lifestyle modifications incorporating a healthy diet, physical activity, brain training and health monitoring have proven effective in preventing dementia and related cognitive decline (REF). The Australian‐Multidomain Lifestyle Intervention to reduce dementia risk (AU‐ARROW) is an ongoing 2‐yearintervention, which is the Australian contribution to the World‐Wide FINGERS network. Here we report on preliminary findings of baseline dietary energy and nutrient intakes of AU‐ARROW participants.

**Methods:**

Data from 99 cognitively‐unimpaired adults enrolled in AU‐ARROW were included (60% female; mean age 67.8±5.3 years). Participants self‐administered the validated Cancer Council of Victoria Food Frequency Questionnaire at baseline, which was used to analyse dietary energy and nutrient intake. Nutrient intakes were compared against the Australian National Health and Medical Research Council Recommended Dietary Intakes (RDIs).

**Results:**

Participants’ average energy and protein intakes were 8980±1980 kJ/day and 96.8±22.6 g/day for males, and 8250±2660 kJ/day and 88.1±25.3 g/day for females. Total fat intake (males 101±25.8 g/day, females 98.9±37.0 g/day) comprised approximately 31% saturated fat, 45% monounsaturated fat and 18% polyunsaturated fat. RDIs were met by participants for most vitamins and minerals, with exceptions for sodium, zinc, calcium, vitamin A, thiamine, riboflavin, vitamin D and iodine. Males exhibited higher sodium intake (2410±588 mg/day) than the Recommended Upper Level of Intake (2300 mg/day), and lower zinc (10.9±2.7 mg/day) and vitamin A (797±310 mg/day) intake than the RDI (Zinc 14 mg/day, vitamin A 900 mg/day). Females exhibited lower thiamine (0.8±0.6 mg/day) and riboflavin (1.0±0.7 mg/day) intake than the RDI (1.1 mg/day for both). Both genders exhibited lower calcium intake (males 856±264 mg/day, females 860±345 mg/day), and iodine (males 123±40.1 µg/day, females 125±54.9 µg/day) than the RDI (calcium: males 1000 mg/day, females 1300 mg/day, iodine: 150 µg/day for both).

**Conclusion:**

Habitual macro‐nutrient intakes of AU‐ARROW participants largely meet the RDI for Australians, except for higher saturated fat and lower calcium and iodine intakes. The Australian Health Survey:2011‐2012 has also reported similar concerns on Australians’ usual nutrient intakes. Attention to a healthy diet is crucial for optimal saturated fat, vitamin, and mineral levels. These baseline data identify deficiencies in the Australian diet which hopefully will be corrected following dietary‐intervention of the AU‐ARROW participants.

